# Differential Expression Profiles of lncRNA Following LPS-Induced Inflammation in Bovine Mammary Epithelial Cells

**DOI:** 10.3389/fvets.2021.758488

**Published:** 2021-10-29

**Authors:** Jin-Peng Wang, Qi-Chao Hu, Jian Yang, Zhuo-Ma Luoreng, Xing-Ping Wang, Yun Ma, Da-Wei Wei

**Affiliations:** ^1^School of Agriculture, Ningxia University, Yinchuan, China; ^2^Key Laboratory of Ruminant Molecular Cell Breeding, Ningxia Hui Autonomous Region, Yinchuan, China

**Keywords:** dairy cow, lncRNA, bovine mammary epithelial cells, mastitis, RNA-seq

## Abstract

Bovine mastitis is an inflammatory response of mammary glands caused by pathogenic microorganisms such as *Escherichia coli* (*E. coli*). As a key virulence factor of *E. coli*, lipopolysaccharide (LPS) triggers innate immune responses via activation of the toll-like-receptor 4 (TLR4) signaling pathway. However, the molecular regulatory network of LPS-induced bovine mastitis has yet to be fully mapped. In this study, bovine mammary epithelial cell lines MAC-T were exposed to LPS for 0, 6 and 12 h to assess the expression profiles of long non-coding RNAs (lncRNAs) using RNA-seq. Differentially expressed lncRNAs (DElncRNAs) were filtered out of the raw data for subsequent analyses. A total of 2,257 lncRNAs, including 210 annotated and 2047 novel lncRNAs were detected in all samples. A large proportion of lncRNAs were present in a high abundance, and 112 DElncRNAs were screened out at different time points. Compared with 0 h, there were 22 up- and 25 down-regulated lncRNAs in the 6 h of post-infection (hpi) group, and 27 up- and 22 down-regulated lncRNAs in the 12 hpi group. Compared with the 6 hpi group, 32 lncRNAs were up-regulated and 25 lncRNAs were down-regulated in the 12 hpi group. These DElncRNAs are involved in the regulation of a variety of immune-related processes including inflammatory responses bMECs exposed to LPS. Furthermore, lncRNA TCONS_00039271 and TCONS_00139850 were respectively significance down- and up-regulated, and their target genes involve in regulating inflammation-related signaling pathways (i.e.,Notch, NF-κB, MAPK, PI3K-Akt and mTOR signaling pathway), thereby regulating the occurrence and development of *E. coli* mastitis. This study provides a resource for lncRNA research on the molecular regulation of bovine mastitis

## Introduction

Mastitis is one of the most common diseases in dairy cows worldwide, causing damage to mammary tissue, reduces milk production, decreases the quality of dairy products, and increases mortality and culling rates (1, 2). Although mastitis is a common disease, it is both expensive and difficult to treat, resulting in huge losses in the dairy industry (3–5). Mammary gland inflammation is primarily a response to an infectious process (2, 6, 7), with *Escherichia coli* (*E. coli*) being a prominent etiologic agent (7, 8). When *E. coli* infects host cells, lipopolysaccharide (LPS, a pathogen-associated molecular pattern) (3, 9) is recognized by toll-like-receptor 4 (TLR4) which triggers immune responses, thus acting as an early signal of pathogenic microbial infection (8, 10). This leads to activation of the nuclear factor kappa B (NF-κB) signaling pathway and induction of the expression of several pro-inflammatory genes, such as interleukin-1 (IL-1), interleukin-6 (IL-6), and tumor necrosis factor-α (TNF-α) (11).

With the advances and popularization in omics technologies in recent decades, long non-coding RNA (lncRNA) and its functions have been mined. lncRNAs, a novel class of transcripts over 200 nucleotides (nt) in length, are located in the nucleus or cytoplasm and cannot encode proteins (12–14). However, it has been suggested that some lncRNAs can form small peptides which affect biological functions (13, 15), participate in inflammatory responses, and regulate the occurrence and development of a variety of diseases (11, 16, 17). At present, an increasing number of studies have shown that lncRNAs also exert significant regulatory effects in the process of bovine mastitis (16, 18, 19). However, few studies on the regulatory mechanisms in dairy cows have been reported in the literature, and this is a critical shortcoming.

This study aimed to detect the expression profile of lncRNAs and elucidate differentially expressed lncRNAs (DElncRNAs) associated with LPS-induced inflammation in bovine mammary epithelial cells (bMECs) in *vitro*. It is hoped that this study can establish a potential molecular regulatory mechanism of dairy cow mastitis to serve as a reference for dairy cattle genetic breeding and to promote the improvement of dairy cow breeding.

## Materials and Methods

### Sample Collection and *in vitro* Mastitis Model

The previously frozen bovine mammary epithelial cell lines MAC-T (20) in liquid nitrogen were thawed and cultured in three 6-well plates supplemented with Dubecco's modified Eagle medium/ F12 containing 10% fetal bovine serum (System Biosciences, Mountain View, CA). When bMEC monolayers reached 80% confluence, one 6-well plate cells (0 h) were collected as the control group, and the remaining two 6-well plates were treated with 50 ng/μL LPS to stimulate an inflammatory response (21). The respective 6-well plates were collected at 6 and 12 hpi as the experimental groups.

### RNA Extraction, Library Construction, and Sequencing

Total RNA was extracted from bMECs induced with LPS at 0, 6, and 12 h using TRizol reagent (Takara Biomedical Technology Co., Ltd, Beijing, China) according to the manufacturer's instructions. The quality of RNA was evaluated by Agarose Gel Electrophoresis, NanoDrop (NanoDrop Technologies, Wilmington, DE, USA), and Agilent 2,100 Bioanalyzer (Agilent, Santa Clara, CA, USA) to ensure the use of qualified samples for sequencing. Subsequently, ribosomal RNA (rRNA) was depleted from total RNA using the Epicentre Ribo-zero™ (rRNA) Removal Kit (Epicentre, Madsion, WI, USA). Preparation of the RNA sequencing library construction was prepared as previously reported (22), that is, RNA library for RNA-seq was prepared as rRNA depletion and stranded method. Then the library concentration was preliminarily tested using Qubit 2.0, and was adjusted to 1.0 ng/μL. Afterwards, the Agilent 2,100 Bioanalyzer (Agilent, Santa Clara, CA, USA)was used to measure insert sizes of the acquired library. To ensure library quality, quantitative real-time PCR (qRT-PCR) was used to accurately determine the library effective concentration (>3 nM). After qualification of the library, the Illumina PE150 platform was used for sequencing according to the library effective concentration and the pooling of data output requirements.

### Quality Control and Mapping of Sequencing Data

The raw data (raw reads) containing a small number of reads with sequencing adapters or with low sequencing quality was obtained by RNA-seq. For the reliability of the data analysis, it was necessary to filter the raw data using the following parameters: (1) reads with adapter were removed; (2) unidentifiable base information reads with a ratio of > 0.002 were removed; (3) the paired reads were removed when the number of low-mass bases in single-end read was more than 50% of the read length ratio. The obtained clean reads were mapped to the internal reference genome (Bos taurus Ensembl 97) by using Hisat2 (23).

### lncRNA Identification

In this study, all the transcripts were merged using Cuffmerge software. LncRNAs were identified according to their structural and functional characteristics. The criteria used to identify the lncRNAs were as follows: (1) transcripts with exon numbers ≥ 2 and lengths > 200 bp were selected; (2) transcript fragments per kilobase per million reads (FPKM) > 0.5 were selected; (3) transcripts that overlap with the database annotation exon regions were identified using the Cuffcompare software (v2.2.1); (4) candidate data sets of novel lncRNAs were obtained through potential coding identification. Then, the final identification and naming were performed to obtain the novel lncRNAs of this analysis according to their loci relative to the coding genes (24).

### Quantification of lncRNA Expression Levels and Differential Expression Analysis

Quantification of lncRNAs was performed using the StringTie software (v1.3.3). The expression level of each transcript was calculated according to the frequency of clean reads. The data were subsequently normalized to the FPKM (25), using the following formula:


$$\begin{array}{l} \text{FPKM}=\text{cDNA Fragments}/[\text{Mapped Fragments }(\text{millions})\\ \;\times \text{ Transcript Length}\;(\text{kb})]. \end{array}$$

Differential expression analyses between two groups were conducted using the Cuffdiff software (v2.1.1). The DElncRNAs were identified using |${\text{log}}_{2}^{\left(\text{FoldChange}\right)}$| > 1 and *P* < 0.05 as standard.

### Validation of RNA-Seq by qRT-PCR

To verify the sequencing results, 10 DElncRNAs were randomly selected to detect their relative expression levels by qRT-PCR. Total RNA extracted by using the Trizol reagent was reverse-transcribed into cDNA using the PrimeScriptTM RT reagent Kit with gDNA Eraser (Takara Biomedical Technology Co., Ltd, Beijing, China) according to the manufacturer's instructions. The qRT-PCR was performed using 2 × M5 HiPer SYBR Premix Es Taq (with Til RNaseH) kit (Mei5 Biotechnology, Co., Ltd, Beijing, China) on a Bio-Rad CFX96 qRT-PCR system (Bio-Rad, Hercules, CA, United States) to analyze the relative expression level of DElncRNAs. The qRT-PCR reaction consisted of the following components: 10.0 μL 2 × M5 HiPer SYBR Premix Es Taq (with Til RNaseH), 0.4 μL Forward Primer, 0.4 μL Reverse Prime, 1.5 μL cDNA template and 7.7 μL RNase-Free ddH2O. The qRT-PCR amplification conditions were as follows: at 95 °C for 30 s, followed by 40 cycles of denaturation at 95 °C for 5 s followed by annealing/extension at 60 °C for 30 s. The qRT-PCR primers for DElncRNAs were designed with Primer Premier 5 (PREMIER Biosoft international, Palo Alto, CA, USA). The primer sequences are presented in Supplementary Table 1. For each sample, 3 biological replicates were amplified. *Glyceraldehyde-3-phosphate dehydrogenase* (*GAPDH*) and *ribosomal protein S18* (*RPS18*) genes were used as internal references (26), and the relative expression of DElncRNAs was calculated using the 2^−ΔΔCT^ method.

The qRT-PCR and RNA-seq data were compared with each other. When the expression trends of qRT-PCR and RNA-seq were consistent, the DElncRNA identification was considered to be reliable.

### Target Gene Prediction and Functional Analysis of DElncRNAs

To explore the potential function of DElncRNAs, target genes of the DElncRNAs were predicted as being either cis-acting and trans-acting. For cis-acting target (co-location) prediction, genes that co-localized to within 100 kb up-or down-stream of a lncRNA were considered to be a cis-acting target (27). For trans-acting target (co-expression) prediction, the Pearson's correlation coefficients between the coding genes and lncRNAs were calculated and analyzed for the identification of trans-acting regulatory elements. This analysis required the sample size was > 5, and a Pearson's correlation coefficient > 0.95 for a gene to be considered a target of the lncRNA (28). Then, Gene Ontology (GO) functional enrichment analysis of target genes of DElncRNAs was implemented by the cluster Profiler R package (R-3.2.4), in which gene length bias was corrected (29).

### Statistical Analysis

All experiments were repeated at least 3 times. Significant differences were analyzed using Student's *t*-test, and *P* < 0.05 was considered to be a significant difference and *P* < 0.01 was extremely significant. The experimental data were presented as mean ± SEM.

## Results

### RNA-Seq of lncRNAs

Nine lncRNA libraries were sequenced using the Illumina PE150 to acquire raw data for quality control, and to extract high-quality clean reads (Table 1). In addition, the Hisat2 software (v2.0.5) was used to compare the clean reads to the reference genome for statistical analysis. For each sample, the clean bases (G) should be ≥ 11.29 G and the Q30 (%) should be ≥ 92.78% to ensure the reliability of results obtained from subsequent analyses. Additional details are provided in Table 1.

**Table 1:** Summary of lncRNA sequencing data.

**hpi**	**Sample name**	**Raw reads**	**Clean bases (G)**	**Q30 (%)**	**Mapped reads**	**Mapped ratio**
0	L_0h_1	91,024,024	13.09	93.35	62,760,360	90.47 %
	L_0h_2	78,017,162	11.29	92.78	53,156,908	90.99 %
	L_0h_3	84,476,878	12.52	94.31	71,165,495	94.05 %
6	L_6h_1	85,070,490	12.57	94.32	40,193,408	92.15 %
	L_6h_2	87,348,140	12.94	93.84	39,633,453	94.27 %
	L_6h_3	91,954,694	13.63	94.23	43,586,453	94.65 %
12	L_12h_1	80,172,386	11.92	94.14	39,818,498	94.39 %
	L_12h_2	90,313,872	13.44	93.84	51,328,167	95.21 %
	L_12h_3	81,278,346	12.09	94.40	45,238,761	95.07 %

### Identification and Classification of lncRNAs

**Figure 1:**
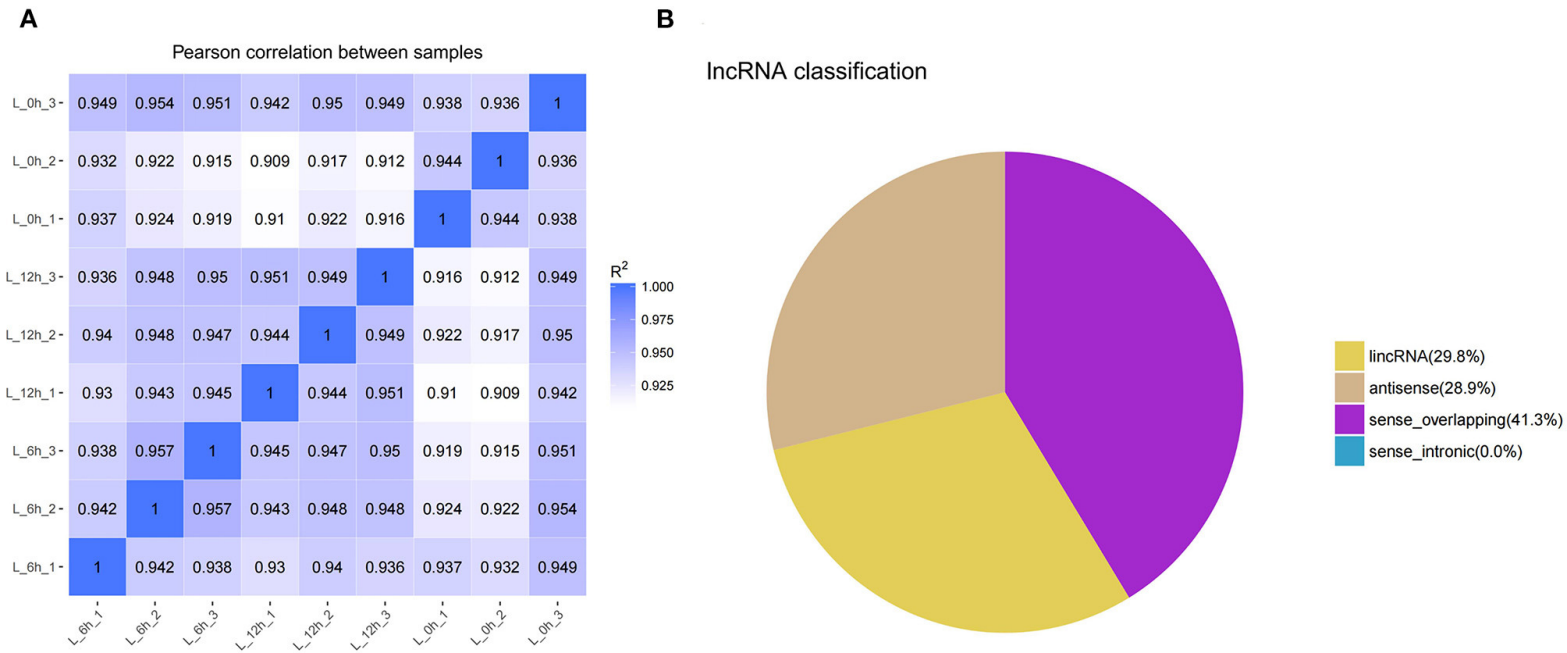
Identification and classification of lncRNA detected in bMECs. **(A)** Pearson's correlation coefficients between samples. **(B)** The types of novel lncRNAs. L_0h is presented that MAC-T were exposed to LPS for 0 h (0 h); L_6h is presented as 6 hpi; L_12h is presented as 12 hpi. (similarly hereinafter).

In this study, the correlation of expression levels between samples was determined to ensure the confidence and rationality of the experiment (Figure 1A). Through analysis of the sequencing data, a total of 2,257 lncRNAs were identified, including 210 annotated and 2,047 novel. Novel lncRNAs were classified as intergenic lncRNA (lincRNA, 29.8%), antisense lncRNA (antisense, 28.9%), and sense overlapping (41.3%) (Figure 1B).

### Profile of lncRNA Expression in LPS Treated bMECs

**Figure 2:**
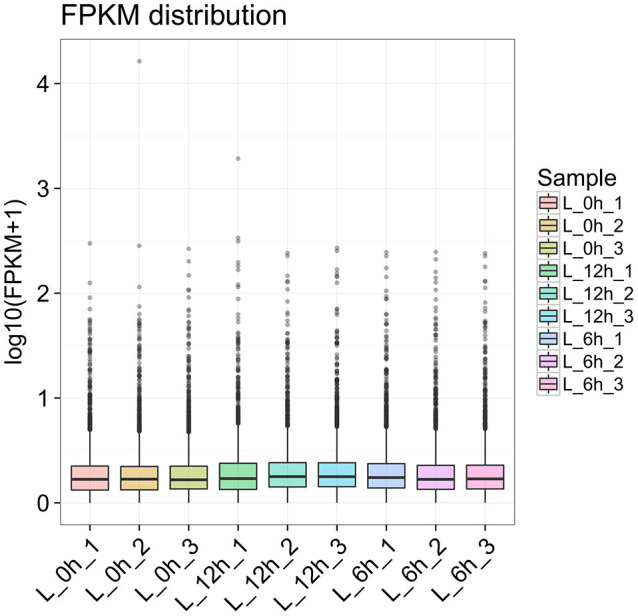
Box-plot diagram of lncRNA expression of all samples at 0 h, 6, and 12 hpi.

To further detect the expression of lncRNA in LPS treated bMECs, cells were incubated for 0, 6, and 12 h following treatment for high-throughput sequencing. In each sample, box-plot diagrams were constructed based on FPKM to observe the expression symmetry and distribution of all lncRNAs. The results showed that the distribution of each box-plot was flat (Figure 2), which indicated that the expression distribution of lncRNA in each sample was correct. At the same time, it can be seen that the distribution of lncRNA expression levels in samples between groups were similar, but small differences were observed.

### Differential Expression Analysis of lncRNA

**Figure 3:**
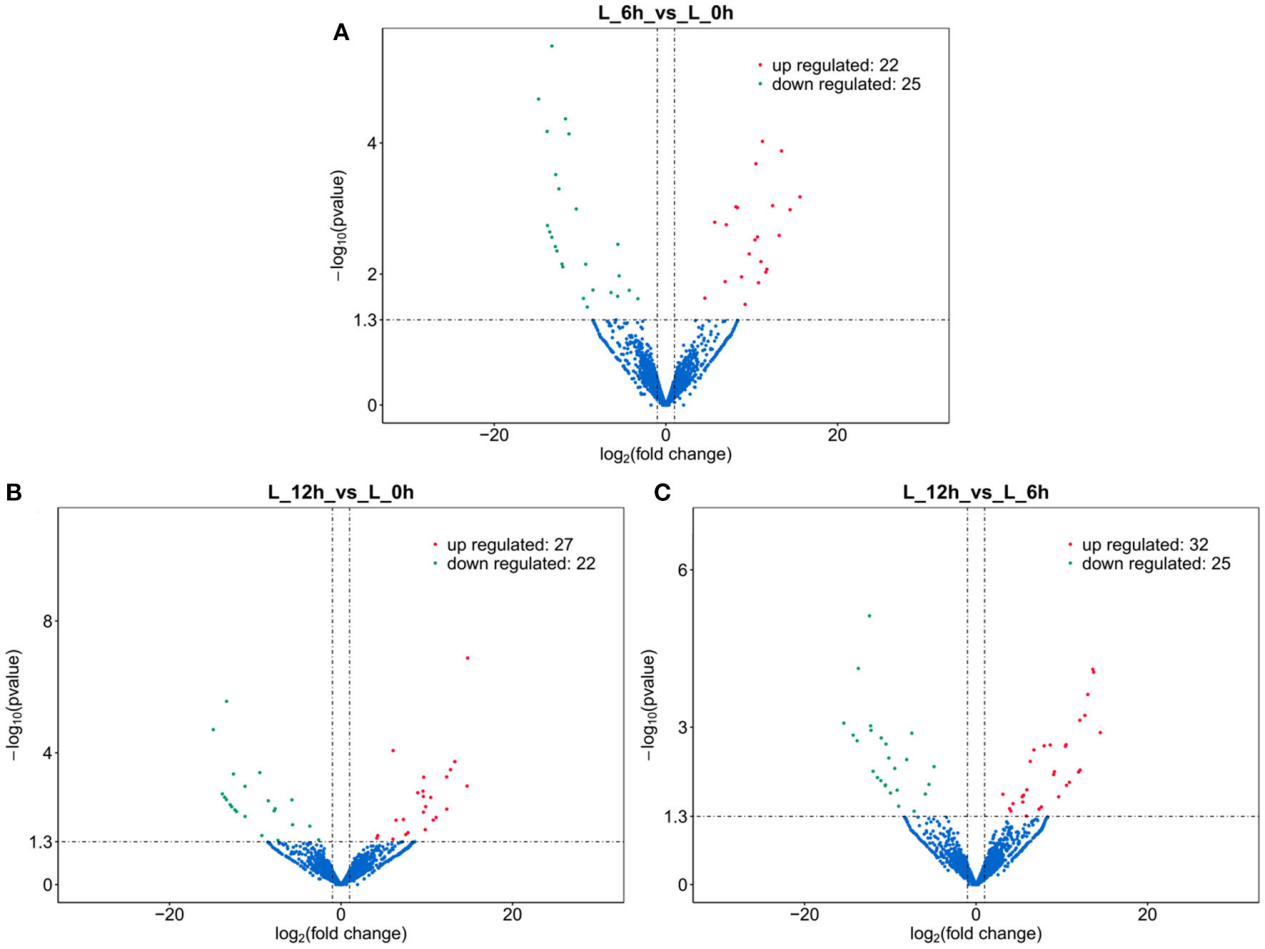
Volcano plot of DElncRNA. **(A)** 6 hpi vs. 0 h. **(B)** 12 hpi vs. 0 h. **(C)** 12 hpi vs. 6 hpi.

**Figure 4:**
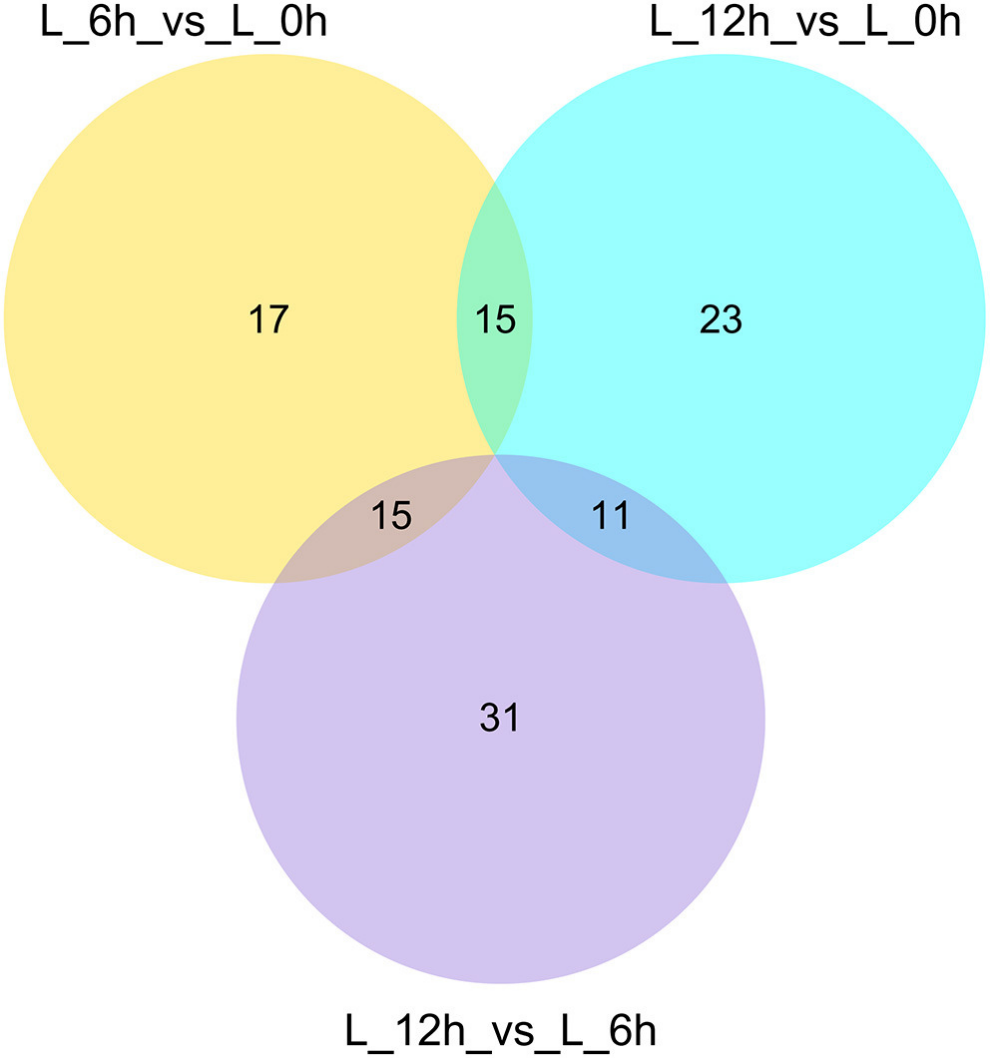
Venn diagrams of DElncRNAs in response to different treatments.

A total of 112 DElncRNAs were observed between the 3 experimental groups. Relative to the 0 h group, there were 22 up- and 25 down-regulated DElncRNAs in the 6 hpi group (Supplementary Table 2; Figure 3A), and 27 up- and 22 down-regulated DElncRNAs in the 12 hpi group (Supplementary Table 3; Figure 3B). Compared with the 6 hpi group, 32 up- and 25 down-regulated DElncRNAs were observed in the 12 hpi group (Supplementary Table 4; Figure 3C). Moreover, among the 3 groups, a total of 17, 23 and 31 specific DElncRNAs were observed in 6 vs. 0 hpi, 12 vs. 0 hpi, and 12 vs. 6 hpi groups by Venn diagram analyses, respectively (Figure 4). It is worth noting that lncRNA TCONS_00039271 was highly expressed and significantly down-regulated (${\text{log}}_{2}^{\left(\text{FoldChange}\right)}$ = −1.9) at 12 hpi relative to 0 h. Meanwhile, lncRNA TCONS_00139850 was not detected in the 0 h and 6 hpi group and was significantly up-regulated (${\text{log}}_{2}^{\left(\text{FoldChange}\right)}$ = 15.94) in the 0 h and 12 hpi group. The results indicated that these DElncRNA may play an important role in the regulation of the inflammatory response following LPS treatment of bMECs.

### Validation of RNA-Seq Results by qRT-PCR

**Figure 5:**
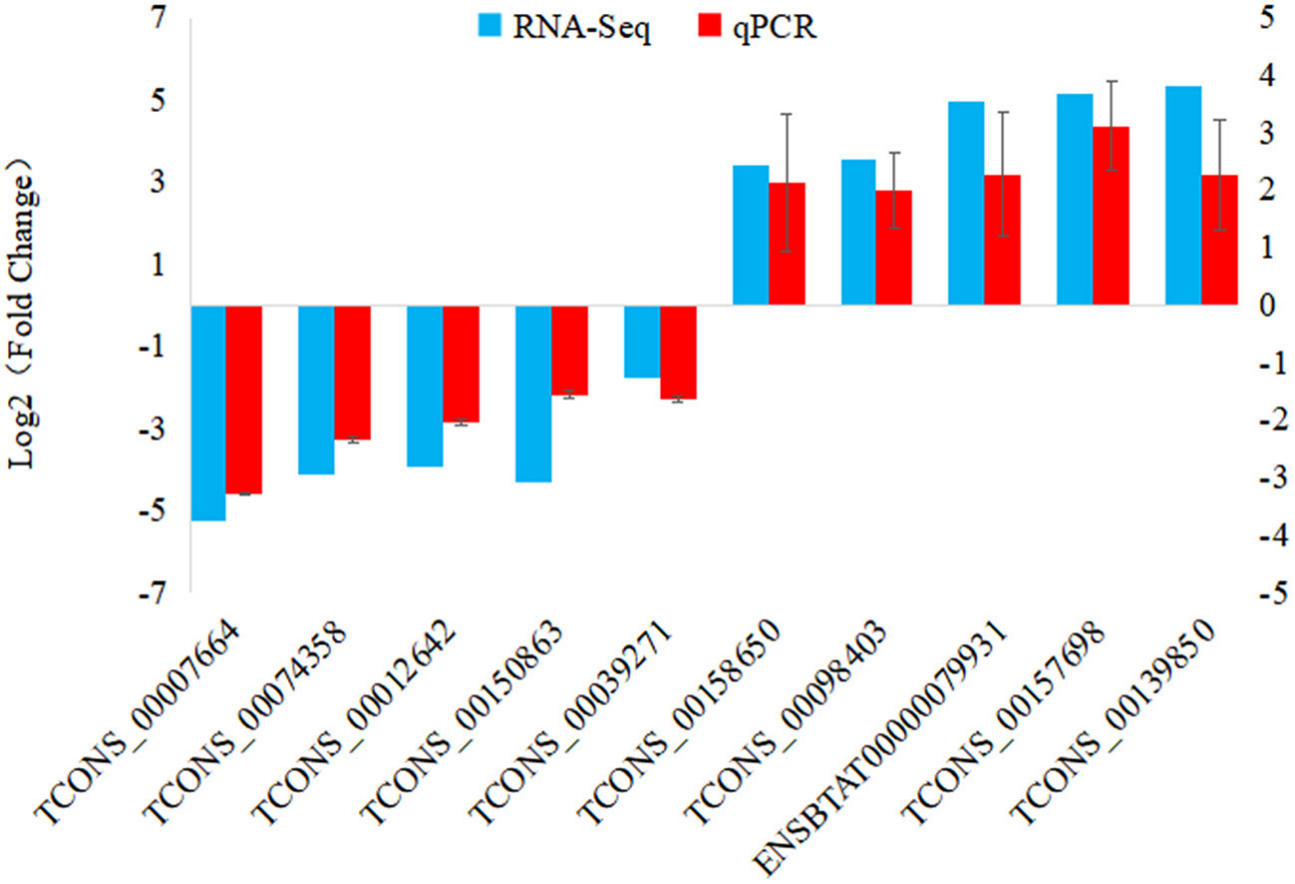
qRT-PCR validation of DElncRNAs. 10 DElncRNAs were used for qRT-PCR validation, with TCONS_00074358 from 6 hpi VS. 0 h comparison; TCONS_00012642, TCONS_00150863, TCONS_00039271, ENSBTAT00000079931, TCONS_00157698 and TCONS_00139850 from 12 hpi VS. 0 h comparison; TCONS_00007664, TCONS_00158650 and TCONS_00098403 from 12 hpi VS. 6 hpi comparison.

To verify the accuracy and reliability of the RNA-seq data, a total of 10 DElncRNAs were randomly selected for expression level analysis by qRT-PCR. Comparing the qRT-PCR results with the RNA-seq results, as shown in Figure 5, the data obtained by the two methods yielded similar patterns, indicating that the screened DElncRNAs based on RNA-seq were reliable.

### Target Gene Prediction and Functional Analysis of DElncRNAs

**Figure 6:**
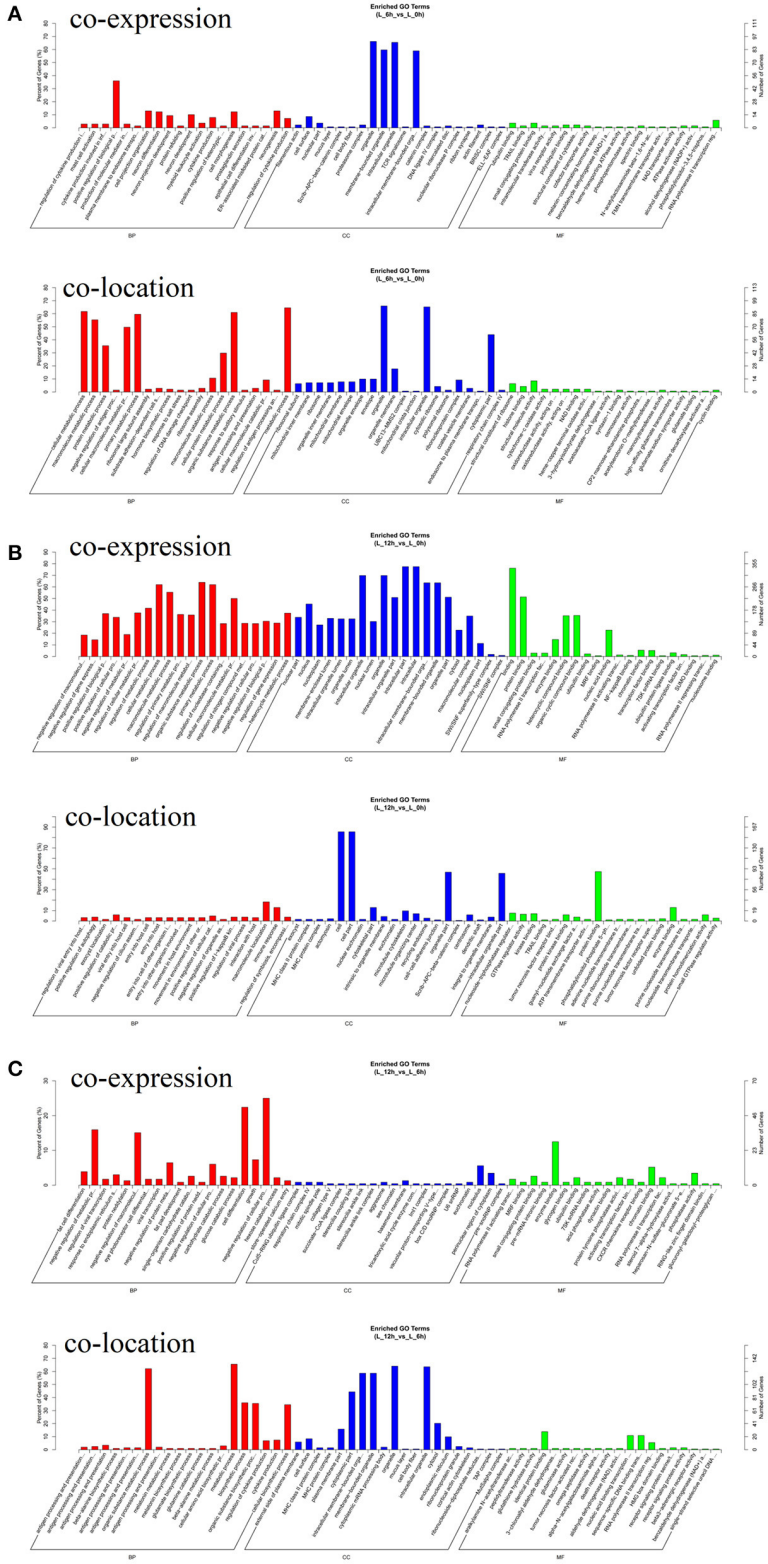
GO enrichment analysis of target genes for DElncRNA. **(A)** Functional enrichment analysis of DElncRNAs at 6 hpi compared to 0 h. **(B)** Functional enrichment analysis of DElncRNAs at 12 hpi compared to 0 h. **(C)** Functional enrichment analysis of DElncRNAs at 12 hpi compared to 6 hpi.

To further investigate the physiological and molecular functions of DElncRNAs on inflammatory reactions in bMECs, or more generally, the pathogenesis of bovine mastitis, possible target genes of DElncRNAs were predicted via co-location and co-expression methods (27, 28). The prediction results of target genes showed that 112 DElncRNAs may have 1,042 target genes. The results of GO enrichment showed that these target genes were involved in the regulation of various biological processes such as inflammatory response, immune response, stress response and regulation of cytokine production (Figure 6). Hence, it can be speculated that these DElncRNAs may participate in the inflammatory response by regulating their target genes during LPS-induced inflammatory response of bMECs.

**Figure 7:**
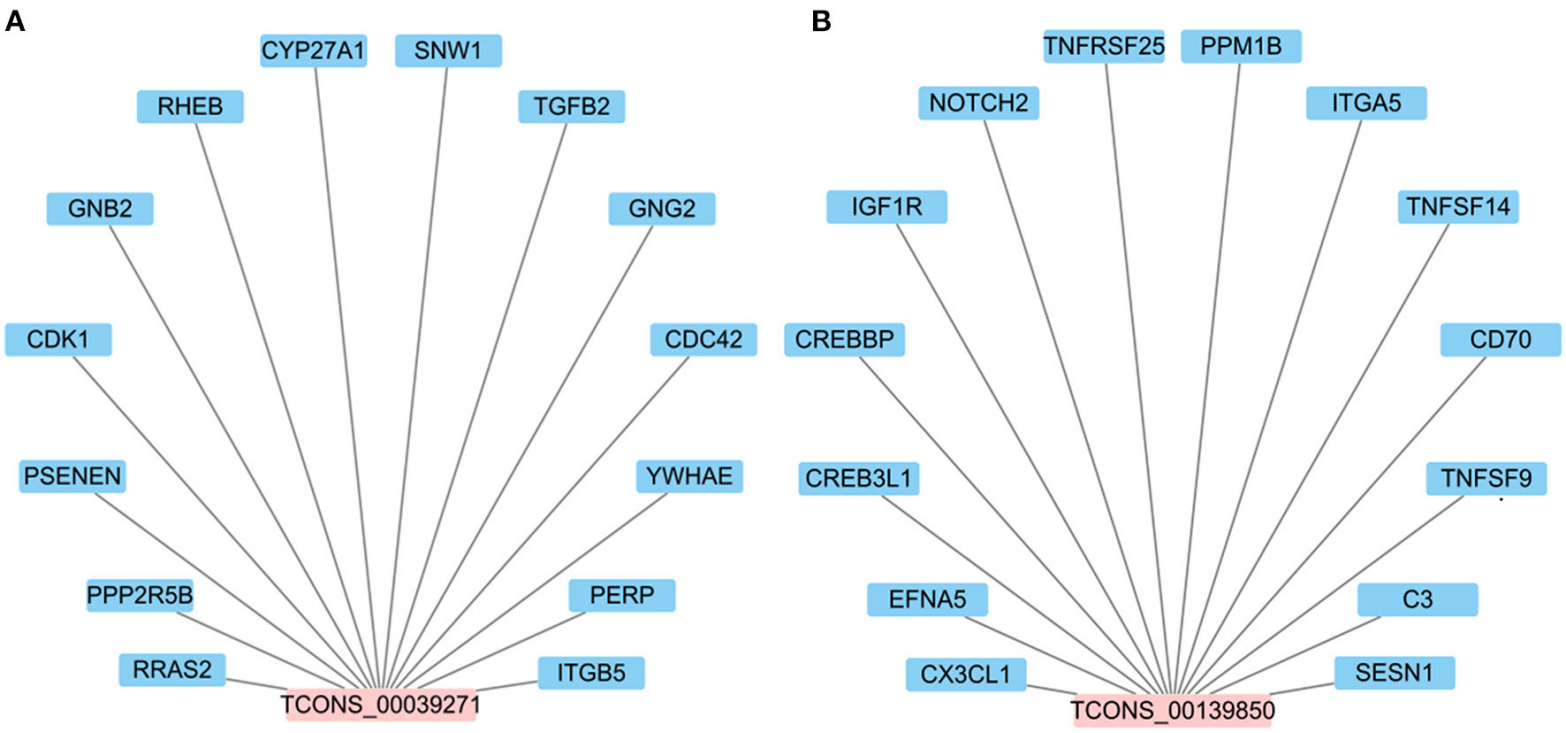
The interaction network between candidate DElncRNAs. **(A)** The potential target genes of lncRNA TCONS_00039271. **(B)** The potential target genes of lncRNA TCONS_00139850.

It is worth noting that TCONS_00039271 and TCONS_00139850 were stably down- and up-regulated, respectively. Constructing the network between lncRNAs and target genes indicated that each DElncRNA possibly targets approximately14 genes (such as *NOTCH2, TNFSF14, CDC42, GNG2, RHEB* and *ITGA5*) (Figure 7; Supplementary Table 5). Previous studies have shown that these target genes involved in inflammatory pathways including the Notch (30), NF-κB (31). MAPK (32), chemokine (33), mTOR (34) and PI3K-Akt signaling pathways (34, 35). In addition, Li et al. (30) and Zhao et al. (36) respectively reported that lncRNA DLEU2 and lncRNA LINC01410 can elevate the expression of *NOTCH2* to stimulate Notch signaling pathway, thereby boosting cell proliferation and hindering cell apoptosis. Taken together, the data suggest that lncRNA TCONS_00139850 and TCONS_00039271 might play a role in regulating the inflammatory response of bMECs via their target genes (Figure 7; Supplementary Table 5). As such, these lncRNAs and their target genes will be the focus of future research on the molecular mechanism of mammary inflammation-related lncRNAs.

## Discussion

Cow mastitis is an inflammatory disease of mammary tissue. Often, it is caused by the invasion and proliferation of pathogenic microorganisms, physical damage, chemical stimulation and the cow's immune response (1, 2, 37). However, among these causes, *E. coli* is the most common culprit (38, 39). When *E. coli* successfully penetrate the mammary gland through the physical barrier of the teat end, it causes an inflammatory response in the mammary gland (38, 40). This colonization leads to rapid changes in the expression levels of immune-related genes, resulting in the inflammatory process (41, 42). In addition, it has been reported that lncRNAs play an important role in pathogen-induced acute and chronic inflammation by regulating the expression of specific genes (43). Therefore, it is extremely necessary to study the DElncRNAs in bovine inflammatory or disease states.

In the past decade, based on the rapid development of high-throughput sequencing technology, an increasing number of studies have reported that lncRNAs are widely present in eukaryotes, especially mammals, and that lncRNAs play an important role in the pathogenesis of many diseases such as cancer and immunodeficiency (44–46). In recent years, a large number of animal and human DElncRNAs have been screened for their possible regulatory functions (47, 48). However, as far as bovine mastitis is concerned, few studies have examined the role of DElncRNAs in bovine mastitis using RNA-seq (16, 18). For example, Wang et al. (16) found 53 DElncRNAs in *E. coli* and *S. aureus*-treated MAC-T cells after 24 h. Wang et al. (18) also identified 21 DElncRNAs from *S. aureus*-treated bMECs after just 2 h. Based on previous reports (7, 14, 16, 42), the expression of lncRNAs in the inflammatory response of bMECs are not always consistent. The reason may be that the process of cow mastitis is a complex process and the changes in expression of many genes, including lncRNA, may be related to the duration of infection. Therefore, RNA-seq was used here to detect the differential expression of lncRNA in bMECs induced by LPS to clarify the role of lncRNA in the process of bovine mastitis. A total of 2,257 lncRNAs and 112 DElncRNAs were observed in bMECs exposed to LPS at 0, 6 and 12 h. By comparing the results of this experiment with the existing literature (16), differences in the type and expression levels of lncRNAs at different times of induction were observed. Thus, it is likely that lncRNAs are involved in the regulation of inflammation and potentially the process of bovine mammary gland inflammation.

To elucidate the potential functions of DElncRNAs, target gene prediction and GO functional enrichment analysis of DElncRNAs revealed that 112 DElncRNAs in LPS-induced bMECs might regulate the expression of 1,042 target genes belonging to several significant signaling pathways. Some such pathways include NF-κB, AMPK, PI3K-Akt, mTOR and MAPK, which participate in the regulation of cytokine production, inflammatory responses and immune responses (31, 32, 34, 35, 49, 50). At the same time, previous studies have shown that the above signaling pathways are closely related to the occurrence, development, and regulation of mastitis (7, 51–53). Additionally, LPS can activate TLR4 and initiate the innate immune and inflammatory response through the activation of NF-κB and MAPK in mammary epithelial cells (54, 55), which induce the expression of pro-inflammatory cytokines IL-6 and TNF-α (56). Liu et al. (57) showed that lncRNA RP11-490M8.1 could reduce LPS-induced pyroptosis via TLR4/NF-κB signaling pathway. Furthermore, Zhao et al. (58) found that lncRNA UCA1 could accelerate cell viability, block apoptosis and function as competitive endogenous RNA (ceRNA) to target TLR4 by sponging miR-499b-5p, thereby alleviating the LPS-challenged cell damage. Based on above results, it was speculated that these DElncRNAs are likely involved in regulating the process of bovine mastitis via their immune-related target genes (7, 59–62).

Based on the data presented here in combination with the literature discussed above, it is clear that further examination of the lncRNA expression profile in mastitis models is needed to clarify molecular mechanisms in regulating the inflammatory process. Unfortunately, there are few reports investigating the role of lncRNAs in the pathogenesis of bovine mastitis. So far, only four lncRNAs, including XIST (7), LRRC75A-AS1 (14), TUB (16) and H19 (42), have been studied. It was reported that lncRNA XIST can significantly inhibit *E. coli* or *S. aureus*-induced inflammatory responses in bMECs through the NF-κB/NLRP3 inflammasome pathway (7). It was observed here that changes in expression of lncRNA TCONS_00039271 and TCONS_00139850 at 0 vs. 12 hpi group were opposite. In order to explore their potential functions, target genes predicted via co-location and co-expression showed that lncRNA TCONS_00039271 might regulate *TGFB2* and *CDC42* (Supplementary Table 5). In mammals, *TGFB1* and *TGFB2* have highly homologous, which can activate the same downstream signaling pathways (63). Increasing studies have showed that *TGF?* can moderate immune and inflammatory responses in mammary gland development (63, 64). It has been reported that *TGFB2* is involved in regulating apoptosis and inflammatory responses in bMECs (65). Meanwhile, Yang et al. (66) showed that highly expressed lncRNA H19 in inflammatory MAC-T cells and bovine mammary tissue can mediate *TGFB2*-induced epithelial mesenchymal transition (EMT) occurance and extracellular matrix (ECM) protein excessive synthesis through the PI3K/AKT signaling pathway to aggravate inflammation. Moreover, the *CDC42* gene also plays an important role in the inflammatory response (67, 68). In addition, for lncRNA TCONS_00139850, a total of 14 potential target genes involved in immunity, such as *CX3CL1* and *NOTCH2* were identified (Supplementary Table 5). Previous studies have reported that *CX3CL1* (69) and *NOTCH2* (70) can participate in inflammatory responses as master regulators. Based on the results of the above analysis, it was concluded that lncRNA TCONS_00039271 and TCONS_00139850 might be involved in the inflammatory response of LPS-initiated mastitis.

In brief, high-throughput sequencing, in concert with previous studies (16), has shown that DElncRNAs might play a crucial role in the regulation of immune and inflammatory responses via regulating their target genes to involve in inflammation-related signaling pathways. Therefore, in-depth exploration and comprehensive studies of the molecular structure and regulatory processes of DElncRNAs will help to further reveal the differences in the development of bovine mastitis.

## Conclusion

Abundant lncRNAs were expressed in LPS-induced bMECs. A total of 112 DElncRNAs were identified following different lengths of exposure to LPS. These DElncRNAs might be involved in the regulation of immune and inflammation responses in *vivo* via several significant signaling pathways including NF-κB, AMPK, PI3K-Akt, mTOR and MAPK signaling pathways. In addition, lncRNA TCONS_00039271 and TCONS_00139850 were stably down- and up-regulated, respectively, and they might act as key regulators of cow mastitis caused by *E. coli*. This study lays a foundation for further research on the molecular regulation of bovine mastitis, and provides a reference for genetic traits desirable to breeding efforts in the control of mastitis in dairy cows.

## Data Availability Statement

The original contributions presented in the study are publicly available. This data can be found here: GEO DATABASE (https://www.ncbi.nlm.nih.gov/geo/query/acc.cgi?acc=GSE181658).

## Author Contributions

J-PW, X-PW, and Z-ML designed the experiments. J-PW and Q-CH completed the experiments. J-PW, X-PW, Z-ML, and JY analyzed the data. J-PW drafted the paper. J-PW, X-PW, Z-ML, YM, and D-WW revised the manuscript. All authors contributed to the article and approved the submitted version.

## Funding

This research was financially supported by the National Natural Science Foundation of China (Grant Nos. 32060749 and 32172709), Project of Ningxia Hui Autonomous Region Key Research and Development Program (Special Talent Introduction) (Grant No. 2019BEB04002), Scientific Research Project of Ningxia Higher Education Institutions (Grant No. NGY2020007), and Research Initiation Project of Talent Introduction of Ningxia University (Grant No. 030900002151).

## Conflict of Interest

The authors declare that the research was conducted in the absence of any commercial or financial relationships that could be construed as a potential conflict of interest.

## Publisher's Note

All claims expressed in this article are solely those of the authors and do not necessarily represent those of their affiliated organizations, or those of the publisher, the editors and the reviewers. Any product that may be evaluated in this article, or claim that may be made by its manufacturer, is not guaranteed or endorsed by the publisher.
